# Anxious and Angry: Emotional Responses to the COVID-19 Threat

**DOI:** 10.3389/fpsyg.2021.676116

**Published:** 2021-08-31

**Authors:** David Abadi, Irene Arnaldo, Agneta Fischer

**Affiliations:** Department of Psychology, University of Amsterdam, Amsterdam, Netherlands

**Keywords:** anger, anxiety, conspiracy mentality, populism, terror management theory (tmt), threat, COVID-19 pandemic, hygiene measures

## Abstract

The current coronavirus disease 2019 (COVID-19) pandemic elicits a vast amount of anxiety. In the current study, we investigated how anxiety related to COVID-19 is associated with support for and compliance with governmental hygiene measures, and how these are influenced by populist attitudes, anger at the government, and conspiracy mentalities. We conducted an online survey in April 2020 in four different countries (Germany, the Netherlands, Spain, and the UK; *N* = 2,031) using a cross-sectional design. Results showed that (1) anxiety related to COVID-19 is associated with conspiracy beliefs, anger at the government, and populist attitudes, and (2) support for and compliance with hygiene measures are both positively predicted by anxiety related to COVID-19; however, (3) support for hygiene measures is also predicted by populist attitudes and negatively by conspiracy mentalities, whereas compliance with hygiene measures is more strongly predicted by anger at transgressors (anger at people transgressing the hygiene measures). Consequently, although anxiety related to COVID-19 concerns the health of individual people, it also has political and social implications: anxiety is associated with an increase in anger, either at transgressors or the government.

## COVID-19 Threat

Previous studies on infection outbreaks and recent ones on the current COVID-19 pandemic have shown that pathogens like the coronavirus not only pose medical health problems, but also elicit a vast amount of anxiety and mental stress (Liu et al., 2020; Robillard et al., 2020; Kruglanski et al., 2021). In line with current theorizing, we argue that anxiety is the central emotion in the response of an individual to the pandemic and, thus, influences how people respond to the threat of becoming contaminated (Van Bavel et al., 2020a,b). We prefer to use the term anxiety rather than fear, although there are many similarities between the two concepts. Fear always has an identifiable object, as one is afraid of something (e.g., Öhman and Rück, 2007), and is related to a specific coping behavior, namely, avoidance (Öhman, 2009). However, when a situation is difficult to control due to uncertainty about the exact nature of a threat, the term “anxiety” is more appropriate. Anxiety refers to a more generalized negative state of mind of foreboding or apprehensive anticipation of future danger, and is thus more applicable to the COVID-19 pandemic.

Anxiety may lead to different types of responses depending on the context, the type of threat, and individual characteristics. For example, on the basis of brain research, researchers have identified a fight, flight, or freeze response (Gray and McNaughton, 2000). In addition, emotion researchers have also found that anxiety or fear does not only lead to the tendencies to run away or avoid threats but also to oppose or attack the threat (depending on the perceived coping potential of the individual; Frijda, 1986; Smith and Lazarus, 1990). Here, we identify two different and seemingly opposite responses, namely, avoiding the threat (by complying with hygiene rules) and fighting the threat (by showing anger at the government or supporting populist attitudes or by conspiracy mentalities). The question we examine is how these different anxiety responses relate to each other, and whether populist attitudes and conspiracy mentalities may affect how an individual supports or complies with hygiene rules.

### Anxiety About COVID-19

Anxiety, more than other negative emotions such as anger or sadness, is characterized by the appraisal of uncertainty: one simply does not know what will happen in the (near) future; this is the core characteristic of anxiety (Roseman, 1984; Smith and Ellsworth, 1985). In addition, research on core relational themes, which reflect a specific combination of various appraisal dimensions, has shown that the core relational theme of fear and anxiety is danger or threat. This implies that the situation is appraised as motivationally relevant, incongruent with the goals of an individual, and characterized by uncertainty and a weak ability to adjust to the threat (Smith and Ellsworth, 1985). The latter two appraisals are especially uncharacteristic for anger or sadness, in which case one knows what one is angry or sad about. There are different ways to cope with emotions (Smith and Lazarus, 1990), but a common element in negative emotions is that people are generally motivated to downregulate negative emotions (e.g., Larsen, 2000) or reduce uncertainty or ambiguity as much as possible (e.g., van Harreveld et al., 2009). Thus, the more anxious people are about becoming infected with the coronavirus, the more they will be motivated to reduce the resulting anxiety (Dixon-Gordon et al., 2015).

Since COVID-19 not only concerns our health but also our social lives and personal wellbeing, it can be regarded as an existential threat. At the time of our data collection, it was still unknown how exactly the coronavirus spreads, how multiple infections can take place, and whether potential vaccines might help against its different variants. This global lack of scientific knowledge and control over such an existential threat triggered anxiety and uncertainty among people. To date, many studies, for example, in China (Chen et al., 2020; Liu et al., 2020; Tian et al., 2020; Wang et al., 2020; Zhu et al., 2020) and in EU countries (Mazza et al., 2020; Ozamiz-Etxebarria et al., 2020; Pieh et al., 2020; Robillard et al., 2020) have shown that people are experiencing anxiety as a reaction to the COVID-19 pandemic.

Not everyone is equally anxious, however, as anxiety levels have been shown to depend on how one estimates the risk of becoming infected (Xu and Cheng, 2021). This might explain why, in some studies, people with the highest probability of becoming infected are the most anxious, such as the elderly (Hyland et al., 2020), people whose friends or family have become infected, or those who lived close to pandemic flashpoints. For example, the population in the central provinces in China where the COVID-19 pandemic broke out reported more distress than anywhere else across China (Zhao et al., 2020). Therefore, anxiety seems to vary with closeness to perceived sources of infection (see e.g., Cao et al., 2020).

### The Role of Anxiety in Responses to the COVID-19 Threat

Depending on individual characteristics, the nature of the threat, and the specific and broader cultural context in which the threat occurs, fear or anxiety may elicit different types of responses. When the perceived threat is acute (e.g., a fire), our neurobehavioral system can respond with three types of responses: fight, flight, or freeze (Gray and McNaughton, 2000). This is in line with studies on experienced action tendencies, which showed that anxious people reported tendencies to avoid, run away from, or attack the threat (Smith and Ellsworth, 1985; Frijda et al., 1989). In the case of the current COVID-19 threat, we distinguished two types of responses. The first response has the goal of avoiding the threat and thus minimize the risk of becoming contaminated, which is comparable to the flight response. This can be effectuated by taking precautionary measures, such as complying with hygiene measures. We expect that, the more anxious people become, the more they will try to avoid infection by the threatening coronavirus, implying that anxiety should be a positive predictor of the support for and compliance with hygiene measures as an individual will try to protect themselves from becoming infected with the coronavirus (see also Harper et al., 2020). People who show this avoidance response are also likely to become angry at people who do not comply with hygiene measures because these transgressors block the goal of these individuals to keep themselves safe (Roseman, 2018).

Anxiety may also elicit a second response, namely, the fight or “attack” response, which is indicative of anger. Although anxiety and anger are characterized by opposite behavioral tendencies (Carver and Harmon-Jones, 2009), both may result from a negative emotional state (Berkowitz, 1993). Blaming others is also a means of regaining a feeling of control or agency to handle the pandemic situation (e.g., Harmon-Jones, 2003; Fischer and Roseman, 2007). Various theories on intergroup threats (e.g., Stephan and Stephan, 2000; Neuberg and Cottrell, 2002), existential threats (Rosenblatt et al., 1989; Greenberg et al., 1997), or personal threats (Hogg, 2012) have also suggested that anxiety may elicit anger responses. The uncertainty that is characteristic of such threats has broader implications, bringing about threats to the self-esteem, life goals, or social relevance of an individual and thus motivating the restoration of the sense of self and significance in life of these individuals (Kruglanski et al., 2021). One of the ways in which this restoration can take place is by blaming others, who are seen as responsible for the negative outcomes in the lives of these individuals (e.g., Greenberg et al., 1997; van Prooijen, 2019).

Terror management theory (Greenberg et al., 1997) posits that death-related anxiety reminds us of our own mortality salience and the transience of our cultural heritage (Rosenblatt et al., 1989). According to this theory, in order to defend ourselves against such death-related anxiety, we fall back on the cultural, religious, and political beliefs that have been part of our worldviews because such beliefs provide symbolic immortality and transcend the biological reality. This worldview defense hypothesis has been supported by various studies showing that, when people are reminded of their own deaths, they intensify their ideologies (Huddy et al., 2005; Huddy and Feldman, 2011), implying that conservatives become more conservative and liberals more liberal in these situations. However, more recent studies suggest that, when reminded of their own death, people show a shift to the right, with this observation being coined as the conservative shift hypothesis (Jost and Napier, 2011; Kosloff et al., 2016; Pyszczynski et al., 2021). Thus, people under threat are more inclined to embrace conservative than liberal political views and tend more to black-and-white thinking about their social worlds. Both hypotheses are supported by a meta-analysis (Kosloff et al., 2016), and the seemingly inconsistent results have been explained by the different ways in which conservatism and other concepts have been measured. Whatever the exact political effects, both hypotheses support the idea that people under threat fall back to simple, one-dimensional worldviews that are often part of their own history. This is also characteristic of populism and a conspiracy mentality.

### Conspiracy Mentality and Populism

Existential threats also give rise to the making sense of the related threatening events. Conspiracy theories (van Prooijen and Acker, 2015) help to explain impactful events, such as a pandemic, with simplistic, one-sided, and proportionally large causes. These conspiracies are often the result of the deliberate will of a clandestine and powerful group, such as cults, secret organizations, or extraterrestrials (Leman and Cinnirella, 2007; van Prooijen and Douglas, 2017; van Prooijen, 2019). People differ in how susceptible they are to explanations based on conspiracy theories, which is referred to as a conspiracy mentality (Bruder et al., 2013). A conspiracy mentality predicts beliefs in specific conspiracy theories and has been shown to be related with right-wing authoritarianism (Imhoff, 2015; Dyrendal et al., 2021). Research on the relationship between stress, anxiety, and conspiracy beliefs has shown inconsistent results, however. For example, a recent study on the relation between self-reported stress and COVID-19-related conspiracy theories did not provide any significant relation, although it did show a relation with a negative attitude toward the government (Georgiou et al., 2020). In addition, another study showed that feeling a lack of control, but not anxiety, was strongly correlated with conspiracy endorsement (Cavojová et al., 2020). According to the Existential Threat Model of Conspiracies (van Prooijen, 2019) that was recently proposed by van Prooijen, conspiracy theories are mainly endorsed when there are salient, antagonistic out-groups who can be blamed for the existential threat. Such out-groups can be low in power, such as minority groups; instances of which we have seen examples during the current COVID-19 pandemic. Especially early in the pandemic outbreak, anti-Chinese sentiment (*Sinophobia*) increased as the Chinese were accused of spreading the coronavirus (Gover et al., 2020).

Salient out-groups can also those be high in power, such as governments, politicians, or CEOs (Douglas, 2021). Populism is characterized by the society being divided into two homogeneous and antagonistic groups (e.g., Mudde, 2004; Wirth et al., 2016; Schulz et al., 2018; Wirz, 2018). This refers to the opposition between “the pure people” and “the corrupt elites” (*Manichean dichotomy*), which has been described as the essence of populism (e.g., Mudde, 2004; Rodrik, 2020). This antagonistic thinking can also be found in conspiracy theories where “actors join together in secret agreement to try to achieve a hidden goal” (van Prooijen and Acker, 2015). Indeed, previous research has also shown a positive relationship between conspiracy mentalities and populist attitudes (Castanho Silva et al., 2017; Balta et al., 2021; Hameleers, 2021) or political extremism (van Prooijen and Acker, 2015).

Because the governments of most countries in the world have taken measures to keep the COVID-19 pandemic at bay, it seems obvious that they can become the target of the anger of populists, as those with populist beliefs tend to blame their governments for not taking the appropriate measures or being too slow to prevent the spread of the coronavirus. Anger toward the government has also been shown to be a general feature of the populist mindset as a part of the populist “us” vs. “them” (social categorization) rhetoric, particularly by showing anger at the government, who are not “us” (see also Abadi et al., 2020; Huguet-Cabot et al., 2021). Moreover, in times of unpredictable changes (Brown, 2000; Hogg, 2001a,b; Abrams and Hogg, 2010), derogating the out-group (in-group favoritism vs. out-group derogation) is often an immediate result of the perceived threat to the social identity of individuals. Thus, anxiety about the coronavirus may strengthen populist attitudes that, in turn, could in fact result in less support for hygiene measures as they were set by the very same government that has been accused of not properly handling the pandemic (Rico et al., 2017; Salmela and von Scheve, 2017; Abadi et al., 2020).

Applying these insights to the COVID-19 pandemic, we assume that the coronavirus causes mortality salience. Anxious individuals may show two different types of responses: avoiding the threat by complying with hygiene measures or fighting the threat, either by showing anger at the government and adhering to populist mindsets or by denying or trivializing the threat (conspiracy beliefs). There is indeed strong evidence for the ability of anxiety to strengthen populist attitudes, as indicated by the increase of in-group favoritism and out-group hostility (Rosenblatt et al., 1989; Greenberg et al., 1990; Schimel et al., 1999), but also conspiracy beliefs (e.g., Grzesiak-Feldman, 2013; Swami et al., 2016; Hollander, 2018). In addition, anxiety has been shown to predict behavioral changes with regard to personal hygiene and social isolation (Harper et al., 2020). The question of our current study is whether anxiety related to COVID-19 indeed prompts these different responses and whether these responses influence each other. In other words, this study explored if conspiracy mentality and populist attitudes also affect whether people support or comply with hygiene measures.

### Current Study

In the current study, we examined the different implications of anxiety about the coronavirus in four different countries: Germany, the Netherlands, Spain, and the UK. At the time of our data collection, the countries differed in amount of COVID-19-related deaths and the measures being taken by the respective governments, with Spain having the highest number of deaths, followed by the UK. We, therefore, expected anxiety related to COVID-19 to be the highest in Spain^1^. However, because the four countries also have distinct public health laws, socio-economic and political contexts, and implemented different measures at different points in time during the COVID-19 pandemic^2^, it is very difficult to compare the results across the countries. We will, therefore, only include country as a variable when examining the factors influencing anxiety. For the other hypotheses, we will use the aggregated data of the four countries.

We tested the following four hypotheses. First (H1), *Anxiety about Coronavirus* is predicted by factors that reflect the proximity to sources of infection (age, country, and oneself or friends becoming infected with the coronavirus). Second (H2), *Anxiety about Coronavirus* is associated with *Support for* and *Compliance with Hygiene Measures*, and *Anger at Transgressors*, which all suggest a strategy to avoid the threat and keep safe by trying to diminish the likelihood to become infected; *Anxiety about Coronavirus* is also associated with *Populist Attitudes, Anger at Government*, and *Conspiracy Mentality*. Third (H3), *Support for Hygiene Measures* is positively predicted by *Anxiety about Coronavirus* and *Anger at Transgressors* and negatively by *Conspiracy Mentality, Populist Attitudes*, and *Anger at Government*. Fourth (H4), we hypothesize that *Compliance with Hygiene Measures* is mainly predicted by *Anxiety about Coronavirus* and *Anger at Transgressors* but not by *Populist Attitudes, Anger at Government*, or *Conspiracy Mentality*.

## Methods and Design

### Sampling Procedure and Data Collection

We tested our hypotheses in a large-scale study across four European countries. In view of the COVID-19 pandemic, we included a variety of European countries with different public health laws, socio-economic factors, and political cultures. Our country samples included Germany, the Netherlands, Spain, and the UK. Our desired representative sample size amounted to approximately 500 respondents per country, while quotas based on current UN-census data (*United Nations Data Retrieval System*) were set up for age, gender, and geographical region. In the informed consent, respondents were instructed about the purpose of our study, their voluntary participation, and guaranteed privacy based on the General Data Protection Regulation (GDPR). We obtained ethical approval from the Faculty Ethics Review Board of the University of Amsterdam (Number 2020-SP-12035).

### Survey

The survey began with general information about our study and a request for informed consent (see Appendix A). All respondents were required to give informed consent before proceeding with the actual questions. The survey included both existing and newly developed scales^3^. Cronbach's alpha (α) is the most common measure of internal consistency (“reliability”) of survey items; thus, it was used here to determine how reliable our multiple the Likert-scale questions were.^4^

#### Measures

*Anxiety about Coronavirus*. We developed this scale to measure anxiety related to the coronavirus infection, which included three items, such as “I am concerned about the effects of the Coronavirus” and “I am worried that my family may be affected by the Coronavirus.” The three items (using a 10-point Likert-scale from *not at all* to *extremely*) formed a reliable scale (Cronbach's α = 0.81).

*Conspiracy Mentality*. This scale included five items from the existing scale *Conspiracy Mentality Questionnaire* (CMQ; Bruder et al., 2013), such as “I think there are secret organizations that greatly influence political decisions.” Considering the long history of pandemics inciting anti-Semitism and its recent revival (see Brackmann, 2020; Gerstenfeld, 2020; Kofta et al., 2020), we decided to include the item “Jews or Zionists have engineered the coronavirus as a biological weapon in order to dominate the world”. The six items (using a 7-point Likert-scale from *strongly disagree* to *strongly agree*) formed a reliable scale (Cronbach's α = 0.8).

*Populist Attitudes*. This scale was based on existing items measuring *Populist Attitudes* (Akkerman et al., 2014; Schulz et al., 2018), which was recently revised by Castanho Silva et al. (2020). This scale consisted of three sub-scales, i.e., *People-Centrism* (e.g., “Politicians should always listen closely to the problems of the people”), *Anti-Elitism* (e.g., “The government is pretty much run by a few big interests looking out for themselves”), and *Manichaean Outlook* (e.g., “You can tell if a person is good or bad if you know their political views”). We also created a subscale for *Nativism* by adding three items, such as “The political elites have failed to protect our cultural identity.” The 10 items (using a 7-point Likert-scale from *strongly disagree* to *strongly agree*) formed a reliable scale (Cronbach's α = 0.71).

*Anger at Government*. This scale was developed to measure how respondents evaluated the recent actions of their government concerning the COVID-19 pandemic. It included four items based on previous research on anger by measuring the main anger appraisals (using a 7-point Likert-scale from *strongly disagree* to *strongly agree*), for example, “I think that our government can be blamed for not reacting fast enough to the outbreak of the coronavirus,” which formed a reliable scale (Cronbach's α = 0.81).

*Anger at Transgressors*. We developed this scale to measure how angry respondents were when other people transgressed the hygiene measures set by the government during the COVID-19 pandemic. It included seven items (using a 7-point Likert-scale from *strongly disagree* to *strongly agree*), such as “I think that the main problem is that some people do not follow the rules,” or “I would confront people who transgress the rules,” which formed a reliable scale (Cronbach's α = 0.7).

*Support for Hygiene Measures*. This scale was created to evaluate the level of approval with various hygiene measures imposed during the pandemic. The scale included nine items (using a 7-point Likert-scale from *strongly disagree* to *strongly agree*), such as “Hand washing for 20 seconds more than 5 times a day” and “Wearing a face mask when leaving your house,” and they formed a very reliable scale (Cronbach's α = 0.88).

*Compliance with Hygiene Measures*. This scale included the same nine items as *Support for Hygiene Measures*, with respondents being asked to what extent (using a 7-point Likert-scale from *never* to *always*) they comply with these hygiene measures themselves. The items formed a reliable scale (Cronbach's α = 0.78).

*Infection of Self or Friends*. We asked whether respondents themselves were infected with the Coronavirus (1 = I do not know, 2 = No, 3 = Yes, but not confirmed yet, and 4 = Yes, confirmed) and whether this was the case for their friends or people in their immediate social environment (same categories).

*Demographic Variables and Socio-Economic Status*. We used self-reported data on age, employment status [1 = unemployed, 2 = student, 3 = retired, 4 = (self) employed], gender, marital status (1 = single, 2 = in a relationship, 3 = married, 4 = divorced, 5 = widowed), religiousness (1 = not at all, 10 = extremely), spirituality (1 = not at all, 10 = extremely), and (perceived) subjective socio-economic status [*MacArthur Scale of Subjective Social Status*; Adler et al. (2000); 1 = low, 10 = high]. All survey items can be found in Appendix A.

### Procedure

The survey was first developed in English and then translated into three other languages by the native speakers of our consortium partners before being back-translated into English. In addition, each survey version was individualized based on country specifications, such as country name and culture-specific terms. All translated surveys were uploaded on the *Qualtrics* online survey platform (Version: April 2020) and the survey data were collected after being synchronized with a global research platform (*Cint*) from April 12 to 14, 2020, which provided us a heterogeneous pool of survey respondents across all four countries involved in this study.

A *pre-test* with 50 respondents per country was run to evaluate the survey time taken (on average between 15 and 20 min). It also aimed to assess the clarity of survey items and its suitability to respondents across various countries. Our pre-test results were satisfactory and no further survey revisions were required. In total, our survey resulted in 2,062 respondents, while 31 respondents with missing values were excluded, resulting in 2,031 complete respondents across four European countries.

## Results

### Respondents

Our final sample consisted of 2,031 participants. Only participants who passed the attention check were included in this sample. The characteristics of our sample across four countries included quotas based on current UN-census data, set up for age, gender and geographical region (see Appendix B, Table B1).

### Preliminary Analyses

We first checked all the reliabilities of our main scales per country in order to detect issues with specific items. All scales had Cronbach's alphas (α) similar to the overall reliability and were always higher than 0.6.

### Determinants of Coronavirus Anxiety

Our first hypothesis was that anxiety about COVID-19 is positively predicted by factors that reflect proximity to the sources of infection. We tested this hypothesis by conducting a hierarchical linear regression to examine whether proximity to sources of infection (age, country, and oneself or friends becoming infected) would increase anxiety related to COVID-19 (*Anxiety about Coronavirus*). We controlled for demographic variables, namely, marital status, gender, employment status, education, (perceived) subjective socio-economic status (*MacArthur Scale of Subjective Social Status*; Adler et al., 2000), and religiousness. We added religiousness because we assumed that this may be a more general protection against feeling anxious. We first dummy coded our categorical variables: experience with getting infected or the infection of friends (0 = no, 1 = yes), marital status (0 = no relation, 1 = in relation), employment (0 = unemployed, 1 = employed) and country. We used Spain as the reference category because it had the highest number of COVID-19-related deaths and the strictest governmental hygiene measures at the moment we collected the data. We entered the variables in two blocks: demographic (control) variables in the first step, and age, infection (self and friends), and country in the second step.

Table 1 shows the results of the hierarchical regression analysis. The first model was significant [*F*_(5,2025)_ = 27.619, *p* < 0.0001], showing that gender, marital status, and religiousness contributed to the regression model and implying that women, people in a relationship, and religious people were reported to be more anxious (see also Robillard et al., 2020). The second model, through the addition of factors related to proximity to infection sources, explained another 9% of additional variance [*F*_(11,2030)_ = 33.186, *p* < 0.0001], showing that age, country, and infection of friends further added to the explained variance. The country variables showed that, compared to Spain, which served as the reference group, participants from the three other countries reported significantly lower amounts of *Anxiety about Coronavirus*. Furthermore, the elderly and people whose friends or family had become infected were reported to be more anxious. The individual themselves getting infected did not appear to play a role in the reported anxiety of the respondents.

**Table 1:** Hierarchical regression analysis in two steps for “anxiety about coronavirus” (standardized regression coefficients).

	***B***	***t***	***sr^**2**^***	***R***	***R^**2**^***	**Δ** *R^**2**^*
**Model 1**		**33.793** ^***^		**0.253**	**0.062**	**0.064**
Gender	0.107	4.898^***^	0.105			
Employment	−0.028	−1.228	−0.026			
Marital Status	0.108	4.826^***^	0.104			
Subjective Social Status	−0.001	−0.042	−0.001			
Religiousness	0.194	8.922^***^	0.192			
**Model 2**		28.790^***^		**0.391**	**0.153**	**0.089**
Gender	0.107	5.137^***^	0.105			
Employment	−0.01	−0.476	−0.010			
Marital Status	0.068	3.128^***^	0.064			
Subjective Social Status	0.046	2.019^*^	0.041			
Religiousness	0.179	8.504^***^	0.174			
Age	0.051	2.440^*^	0.050			
Infection (Self)	0.010	0.485	0.010			
Infection (Friends)	0.088	4.055^***^	0.083			
Germany	−0.289	−11.055^***^	−0.226			
UK	−0.104	−4.059^***^	−0.083			
The Netherlands	−0.282	−10.943^***^	−0.240			

*N = 2,031*.

* *p = 0.05*,

*** *p < 0.001*.

These results confirmed the general hypothesis that more exposure to COVID-19 (living in a country with higher infection rates, age, and infection of friends and family) is a positive predictor of anxiety. Some of our control variables also showed some variance in explaining anxiety, which may be interpreted by the fact that women generally tend to report more intense emotions (e.g., Fischer et al., 2004) and people in a relationship may be more concerned about the lives of their loved ones in their immediate social environment. In contrast with our assumption, religiousness did not serve as buffer against the anxiety of individuals, but rather the opposite. People who were religious were more, rather than less, anxious. We further explored whether the positive role of religiousness could be related to different types of religions by checking how religious people reported themselves to be (see Table 2). Clearly, Greek, and Russian Orthodox Christians and Muslims rated themselves highest on religiousness, whereas atheists, agnostics, and non-religious individuals scored the lowest^5^.

**Table 2:** Means and SD of “religiousness,” split for people with various religions.

**Religion**	***M***	***SD***	***N***
Greek-Orthodox	7.70	2.627	10
Muslim	6.98	2.080	87
Russian-Orthodox	6.18	2.786	11
Jewish	5.82	2.481	22
Roman Catholic	5.35	2.419	566
Protestant	5.20	2.554	293
Hindu	5.00	2.530	11
Buddhist	5.07	2.890	15
Spiritual	4.83	3.073	75
Agnostic	2.60	2.078	55
Non-Religious	2.31	2.026	731
Atheist	1.83	1.840	155
Total	3.96	2.805	2,031

In order to test the second hypothesis, we calculated the Pearson correlations between the following variables: *Anxiety about Coronavirus, Support for* and *Compliance with Hygiene Measures, Anger at Transgressors, Populist Attitudes, Anger at Government*, and *Conspiracy Mentality*. As shown in Table 3, all correlations are positive and moderately strong. The strongest correlations were found between *Populist Attitudes, Conspiracy Mentality*, and *Anger at Government*.

**Table 3:** Pearson correlations between scales.

**Scales**	**M (SD)**	**Anxiety about Coronavirus**	**Support for hygiene measures**	**Compliance with hygiene measures**	**Conspiracy mentality**	**Populist attitudes**	**Anger at government**
Anxiety about coronavirus	6.86 (1.96)						
Support for hygiene measures	5.79 (1.02)	0.390^**^					
Compliance with hygiene measures	4.67 (1.21)	0.305^**^	0.327^**^				
Conspiracy mentality	4.34 (1.10)	0.190^**^	0.103^**^	0.258^**^			
Populist attitudes	4.70 (1.43)	0.240^**^	0.306^**^	0.202^**^	0.615^**^		
Anger at government	4.16 (1.43)	0.249^**^	0.138^**^	0.243^**^	0.497^**^	0.436^**^	
Anger at transgressors	4.25 (1.02)	0.329^**^	0.386^**^	0.201^**^	0.214^**^	0.328^**^	0.201^**^

** *p < 0.001*.

In order to test the third hypothesis, namely, that *Support for Hygiene Measures* is positively predicted by *Anxiety about Coronavirus* and *Anger at Transgressors* but negatively by *Conspiracy Mentality, Populist Attitudes*, and *Anger at Government*, we conducted a hierarchical linear regression with *Support for Hygiene Measures* as the dependent variable. We entered *Anxiety about Coronavirus* in the first step, *Populist Attitudes, Anger at Government, Conspiracy Mentality*, and *Anger at Transgressors* in the second step, and country in the third step (see Table 4).

**Table 4:** Hierarchical regression analysis for “support for hygiene measures” in three steps (standardized regression coefficients).

	***β***	***t***	***sr^**2**^***	***R***	***R^**2**^***	**Δ** *R^**2**^*
**Model 1**		**58.037** ^***^		***0.390***	**0.152**	**0.152**
Anxiety about coronavirus	0.310	19.070^***^	0.390			
**Model 2**		**23.882** ^***^		**0.516**	**0.266**	**0.114**
Anxiety about coronavirus	0.281	13.602^***^	0.259			
Populist attitudes	0.262	10.332^***^	0.197			
Anger at government	−0.019	−0.829	−0.016			
Anger at transgressors	0.245	11.696^***^	0.223			
Conspiracy mentality	−0.155	−6.094^***^	−0.116			

*N = 2031*.

*** *p < 0.001*.

The hierarchical regression in Table 4 shows that the first model is significant [*F*_(1,2029)_ = 363.646, *p* < 0.0001]. The second model, through the addition of various types of attitudes and political beliefs, explains an additional 11% of additional variance [*F*_(5,2025)_ = 146.703, *p* < 0.0001], implying that *Populist Attitudes, Anger at Transgressors*, and *Conspiracy Mentality*, but not *Anger at Government*, are significant predictors. As expected, more *Anger at Transgressors* predicted more *Support for Hygiene Measures*, whereas stronger beliefs in *Conspiracy Mentality* predicted less *Support for Hygiene Measures*. Unexpectedly, *Anger at Government* was not significant, whereas *Populist Attitudes* was a positive predictor of *Support for Hygiene Measures*, implying that people who think in terms of “us' vs. “them” are more likely to approve of hygiene measures.

In order to test our fourth hypothesis, which stated that *Compliance with Hygiene Measures* is mainly predicted by *Anxiety about Coronavirus* and *Anger at Transgressors* but not by *Populist Attitudes, Anger at Government*, or *Conspiracy Mentality*, we conducted a similar regression analysis, but with *Compliance with Hygiene Measures* as the dependent variable. Table 5 shows that the first model is significant [*F*_(1,2029)_ = 208.098, *p* < 0.0001], indicating that *Anxiety about Coronavirus* significantly explains the variance in *Compliance with Hygiene Measures*. The second model, through the addition of political beliefs, explained another 5% of additional variance [*F*_(5,2025)_ = 87.126, *p* < 0.0001], showing that *Anger at Government, Anger at Transgressors*, and *Conspiracy Mentality* also added to the explained variance. As expected, *Anxiety about Coronavirus* and *Anger at Transgressors* were positive predictors and *Populist Attitudes* was not; however, *Anger at Government* and *Conspiracy Mentality* were also both positive predictors of *Compliance with Hygiene Measures*.

**Table 5:** Hierarchical regression analysis for “compliance with hygiene measures” in three steps (standardized regression coefficients).

	***β***	**t**	**sr^**2**^**	**R**	**R^**2**^**	**ΔR** ^**2**^
**Model 1**		**36.310** ^***^		**0.328**	**107**	**0.107**
Anxiety about coronavirus	0.305	14.426^***^	0.305			
**Model 2**		**14.729** ^***^		**0.396**	**0.157**	**0.050**
Anxiety about coronavirus	0.229	10.296^***^	0.211			
Populist attitudes	−0.021	−0.760	−0.016			
Anger at government	0.100	4.081^***^	0.077			
Anger at transgressors	0.078	3.448^**^	0.084			
Conspiracy mentality	0.161	5.861^***^	0.120			

*N = 2,031*.

** *p < 0.01*,

*** *p < 0.001*.

## Discussion

We reported the results of an online survey conducted in four European countries in April 2020 only a few weeks after the COVID-19 pandemic had erupted, examining the reported anxiety of individuals in relation to becoming infected with the coronavirus and how this affects their support for and compliance with hygiene measures and anger at the government and conspiracy mentality. Our results, first of all, showed that anxiety about the coronavirus is mostly predicted by factors that reflect proximity to the sources of infection. The elderly, individuals who reported infections in their immediate social environment, and those who lived in countries with high infection rates (Spain) reported the most anxiety. Other demographic variables also played a role. Women and people in intimate relationships reported stronger anxiety than men and people who were single. The fact that women rather than men reported more anxiety may contradict the general idea that the likelihood of becoming a victim would make someone more anxious since hospitalization and death rates among older men is especially higher than among women. However, studies have found that women generally tend to report stronger emotions (Fischer, 2000; Fischer et al., 2004), which is an observation that has been found in other studies on mental stress about infection as well (e.g., Capraro and Barcelo, 2020; Liu et al., 2020). Thus, the subjective appraisal of the threat apparently outweighs the actual likelihood of becoming infected. The finding that people in intimate relationships show more anxiety can be explained by the fact that people generally seem more concerned about the health of their friends and family rather than their own, which is reflected in the finding that anxiety about the infections of others is a stronger predictor for anxiety about the coronavirus than anxiety about becoming infected oneself.

Regarding our main hypotheses, we first found support for the idea that the anxiety of an individual about the coronavirus is positively associated with support for and compliance with hygiene measures and anger at people who transgress the hygiene measures, suggesting that people are foremost trying to avoid becoming infected. However, this did not seem to be the only response, as anxiety is also associated with attacking the threat *via* beliefs that are not health-related: populist attitudes, anger at the government, and adherence to conspiracy theories. In other words, people under threat may fall back to simple, one-dimensional, and extreme worldviews, such as conspiracy theories (see Grzesiak-Feldman, 2013; Swami et al., 2016; Hollander, 2018) or accusations that the actions of the government or the elites are too little, too late to stop the spread of the coronavirus.

Therefore, the more anxious people are, the angrier they may become, regardless if this anger may be directed at their government or at other people. Anger provides a feeling of control where an individual can at least can blame others, which is a state of mind that may be preferred over uncertainty: not knowing what will happen next. Indeed, previous research has shown that people try to feel the emotions that they prefer and seem most useful in specific contexts (e.g., Tamir and Ford, 2012). Other evidence may be found in research on shame and guilt proneness, showing that the tendency to feel ashamed or guilty is related to the tendency to become angry and blame others for negative events (e.g., Tangney et al., 1992). More generally, it has been found that individuals in negative physical or emotional (stressed) states are more likely to become angry and behave aggressively (Berkowitz, 1993). Thus, our finding that anxious people are also more likely to report anger may be the result of a general state of negative arousal due to the COVID-19 crisis. Because of the correlational nature of the data, however, we cannot draw any firm conclusion about the causal effects of anxiety on anger.

Whereas anxiety and anger signal different, even opposite motivational tendencies, namely, avoiding vs. attacking (blaming), there is also evidence suggesting that these emotions can co-occur. For example, it has been argued that people can experience mixed emotions, especially in response to big or ambiguous events (e.g., Ross, 2013; Solomon, 2013; Larsen and McGraw, 2014; Van Rythoven, 2015). Most research on mixed emotions shows that people can feel sad and happy at the same time and in response to the same event; the experience of different negative emotions is even more likely (e.g., Ross, 2013).

Our third hypothesis was that support for hygiene measures enforced by the government is not only predicted by anxiety about the coronavirus, but also negatively by conspiracy mentality, populist attitudes, and anger at the government. As expected, we found that anxiety and anger at transgressors predicted support for hygiene measures. In addition, a conspiracy mentality was a negative predictor, while populist attitudes were a significant positive predictor. Although other studies conducted in the US (e.g., Gollwitzer et al., 2020) suggest that people who supported the Trump administration were less anxious and less supportive of hygiene measures, our data showed a different pattern. In our study, anger at the government was a non-significant predictor, which may be explained by the fact that all governments in our four countries took drastic action, and not least because participants would support hygiene measures independently of the steps their governments took. As expected, support for hygiene measures was negatively predicted by conspiracy mentality, because the beliefs of an individual in clandestine forces and their covert actions in explaining the COVID-19 threat would make the hygiene measures superfluous. Populist attitudes, however, were a positive predictor of support for hygiene measures, which we explained by the fact that anxious people are more prone to populist thinking, which would contribute to their approval of the hygiene measures.

We also found that anxiety, anger at transgressors and the government, and conspiracy mentalities predicted whether individuals themselves complied with the hygiene measures (fourth hypothesis). As expected, the additional explained variance by including populist attitudes, anger at the government, and conspiracy mentalities was less than in the case of support for hygiene measures. Here, anger at the government was a significant positive predictor that may suggest that individuals perceived the measures that were taken as insufficient, and thus made sure to abide by the rules by themselves. This may also explain the positive relation with conspiracy mentality. Assuming that the pandemic would be a global conspiracy, one would not *approve* of any action that restricts the freedom of people, but the salience of illness or death may have increased the anxiety of an individual and overruled their general critical judgment of the government. This is in line with the fact that compliance with hygiene measures was also more heavily predicted by country than support for hygiene measures, presumably because of the variety of pandemic-related restrictions across the four countries.

The differences in significant predictors for approving of and behaving according to the governmental hygiene measures is interesting. The agreement of an individual with certain measures seems more strongly related to their views on the government and belief in conspiracies, whereas following actual measures to prevent contracting an infection seems more strongly related to the anxiety of the individual about becoming infected and their exposure to the coronavirus. This may also explain why country was a stronger positive predictor of compliance than support for hygiene measures. As Spain had the highest death rate and largest number of infections, this context may explain why people supported and complied with measures that would slow down the coronavirus transmission. It may also explain why Spain was consistently different from Germany, the Netherlands, and the UK, especially with regard to compliance with hygiene measures and individual anxiety. On the basis of our present research design and measures, however, we cannot fully address whether other country variables, such as political situation or socio-cultural orientation, may have played a role, so more research is needed to further examine these factors.

## Limitations

One potential limitation of an online survey is the representativeness and quality of the sample. We used quotas based on UN-census data for age, gender, and geographical region. In addition, our demographic data also showed variability in employment status and education level, so we obtained a relatively representative sample. In addition, we added an attention check question, and none of the participants failed this question, indicating that the participants were focused and reliable. Second, we used self-reports, measuring the beliefs, feelings, and behaviors of the respondents. Whereas using self-reports for measuring beliefs and feelings is very common in psychological research, measuring behavior through self-reports may be less accurate and more prone to social desirability effects than, for example, observing behavior. Yet, we were not aware of better methods to collect this type of information during the COVID-19 pandemic lockdown, when people were requested to stay mostly at home. A third limitation concerns the translation of our survey items into four different languages. Words have culture-specific meanings sometimes, and thus were understood slightly differently across the four countries. Our back-translation procedure did not reveal any major issues, however. In addition, we consistently used multiple items to measure each construct; hence, we think that this issue was reduced as much as possible. We also checked the reliabilities of our scales separately for each country, and they were all satisfactory, except for compliance with hygiene measures, the lack of which we explained in the results section.

## Conclusion

Our study shows that anxiety about the coronavirus is not only associated with the motive to avoid the COVID-19 threat by following hygiene measures, but can also lead to anger-related responses, as reflected in populist attitudes, anger at the government, and conspiracy mentalities. In addition to anxiety, these beliefs significantly influence whether one approves and, to a lesser extent, behaves according to governmental hygiene measures to contain the coronavirus. Both support for and compliance with hygiene measures is primarily predicted by the anxiety of individuals and anger at transgressors, but also by the anger individuals have about the threat, suggesting support for the idea that the COVID-19 pandemic causes mortality salience. Thus, anxious people may also become angry and more likely to oppose their government, even though, ultimately, they seem to prefer their own survival over emotionally laden conflicts.

## Data Availability Statement

The datasets presented in this article are not publicly available because based on the GDPR agreements of our H2020 DEMOS research project, they are only available to consortium partners. Requests to access the datasets should be directed to David Abadi, d.r.abadi@uva.nl.

## Ethics Statement

The studies involving human participants were reviewed and approved by Faculty Ethics Review Board of the University of Amsterdam (Number 2020-SP-12035). The patients/participants provided their written informed consent to participate in this study. Written informed consent was obtained from the individual(s) for the publication of any potentially identifiable images or data included in this article.

## Author Contributions

Testing and data collection were performed by DA. Data analysis and interpretation were performed by all authors. DA and AF drafted the manuscript, and all authors provided critical revisions. All authors developed the study concept and contributed to the research design and approved the final version of the manuscript for submission.

## Conflict of Interest

The authors declare that the research was conducted in the absence of any commercial or financial relationships that could be construed as a potential conflict of interest.

## Publisher's Note

All claims expressed in this article are solely those of the authors and do not necessarily represent those of their affiliated organizations, or those of the publisher, the editors and the reviewers. Any product that may be evaluated in this article, or claim that may be made by its manufacturer, is not guaranteed or endorsed by the publisher.

## Footnotes

^1^On April 13, 2020, Spain counted more than 17,000 deaths, while the Netherlands and Germany reported less than 3,000 deaths. In the same week, there was a large increase of coronavirus deaths in the UK, amounting to more than 11,000 deaths.

^2^When our survey was conducted in April 2020, Spanish citizens had already been under full lockdown for 4 weeks (starting in March 14, 2020). On March 16, the prime minister of the Netherlands addressed the nation to inform them about *social distancing* measures that were less strict than in other European countries (“intelligent lockdown”). On the same day, the state of Bavaria in Germany declared a state of emergency, with other German states following soon after. The measures taken in Germany varied per state, thus making it difficult to draw general conclusions on the strictness of policy measures for the whole country. In the UK, the measures became legally enforceable on March 26, and our British sample had, therefore, already experienced the lockdown for over 2 weeks.

^3^In some cases, we used shortened versions of the original scale in order to prevent our Qualtrics survey from becoming too long.

^4^Additional variables were measured but were not reported in the present study. A complete list of measured variables and scales used in our Qualtrics survey [e.g., symbolic and realistic threats, news consumption (headline selection), and threat estimation (material and safety, coronavirus, prosocial behavior, and moral reasoning)] can be found in Appendix A.

^5^We also checked whether the correlation differed across the countries, however this was not the case: in all four countries the correlation was between 0.17 and 0.24. Moreover, feelings of religiousness were not different per country either (*F*_(3, 2030)_ = 1.605, *p* = 0.186).
