# Supplementary material for: Anxious and Angry: Emotional Responses to the COVID-19 Threat
Source: Front Psychol. 2021 Aug 31;12:676116. doi: 10.3389/fpsyg.2021.676116 (PMC8439354; doi:10.3389/fpsyg.2021.676116)
Supplement: Supplementary file 1 [file Data_Sheet_1.docx]

**Appendix A**

Qualtrics Online Survey

A complete list of measured variables and scales used in our Qualtrics survey (e.g., symbolic and realistic threats, news consumption (headline selection), threat estimation (material and safety, Coronavirus, prosocial behavior, moral reasoning) can be found in our Online Appendix:

https://surfdrive.surf.nl/files/index.php/s/XQ7IHwnrkfP6JuK

**Appendix B**

Table B1

Demographic Variables across four countries (N=2031)

| Variables | Categories | Germany | Spain | Netherlands | UK |
| --- | --- | --- | --- | --- | --- |
| Age (%) | 18 - 24 | 8.97 | 9.88 | 9.74 | 12.01 |
|  | 25 - 34 | 22.14 | 22.98 | 22.66 | 23.82 |
|  | 35 - 44 | 23.09 | 34.48 | 25.85 | 23.82 |
|  | 45 – 54 | 26.34 | 19.56 | 21.47 | 21.46 |
|  | 55 - 64 | 17.75 | 12.1 | 18.29 | 17.32 |
|  | 65 - 74 | 1.72 | 1.01 | 1.79 | 1.58 |
|  | 75 - 84 | 0 | 0 | 0.199 | 0 |
| Gender (%) | Male | 50.76 | 50.2 | 52.49 | 47.44 |
|  | Female | 49.05 | 49.8 | 47.52 | 52.56 |
|  | Other | 0.191 | 0 | 0 | 0 |
| Employment (%) | Unemployed | 11.26 | 14.11 | 20.08 | 15.75 |
|  | Student | 6.3 | 6.05 | 5.96 | 2.95 |
|  | Retired | 8.02 | 2.22 | 2.78 | 3.15 |
|  | (Self-)Employed | 74.43 | 77.62 | 71.17 | 78.15 |
| Education (%) | No degree | 2.1 | 0.61 | 1.79 | 5.12 |
|  | High school | 11.64 | 15.52 | 14.51 | 22.05 |
|  | Some university, no degree | 8.59 | 5.65 | 36.18 | 14.76 |
|  | Technical degree | 46.18 | 23.79 | 22.47 | 18.11 |
|  | Bachelor's degree | 13.36 | 38.11 | 8.95 | 26.97 |
|  | Master's degree | 16.79 | 11.9 | 12.33 | 8.47 |
|  | Doctoral degree | 1.34 | 4.44 | 3.78 | 4.53 |
| Religion (%) | Protestant | 23.86 | 1.82 | 12.33 | 19.09 |
|  | Roman-Catholic | 25.76 | 46.17 | 20.48 | 19.49 |
|  | Muslim | 5.73 | 0.81 | 5.77 | 4.73 |
|  | Jewish | 0.76 | 0.61 | 1.59 | 1.38 |
|  | Russian-Orthodox | 0.95 | 0.4 | 0.4 | 0.4 |
|  | Greek-Orthodox | 0.76 | 0 | 0.4 | 0.79 |
|  | Hindu | 0.76 | 0 | 0.4 | 0.98 |
|  | Buddhist | 0.57 | 0.61 | 0.99 | 0.79 |
|  | Agnostic | 1.91 | 6.25 | 0.99 | 1.77 |
|  | Atheist | 6.68 | 14.52 | 3.58 | 5.91 |
|  | Spiritual | 1.91 | 4.64 | 4.97 | 3.35 |
|  | Non-Religious | 30.34 | 24.19 | 48.11 | 41.34 |

**Appendix C**

Post-hoc analyses of differences in *Anxiety about Coronavirus*, differentiated for country, marital status, gender, employment status, and infection of self or friends: Univariate analyses of variance with post hoc tests for each of the key variables show that people from Germany (*M*=6.26, *SD*=2.16) and the Netherlands (M=6.40, SD=1.81) report to feel less anxious than people from the UK (*M*=7.12, *SD*=1.92), who in turn report to be less anxious than people from Spain (*M*=7.69, *SD*=1.53), *F* (4, 2026) = 64.15, *p*<.0001, η_p_^2^=.087. Singles (*M*=6.42, *SD*=2.043) report to be less anxious than people who are in a relationship (*M*=6.88, *SD*=1.88), married (*M*=7.11, *SD*=1.90), or divorced (*M*=7.07, *SD*=1.87), but not different from widowed people (*M*=6.13, *SD*=2.12), *F* (4, 2026) = 12,45, *p*<.0001, η_p_^2^=.024). People without a partner thus seem less anxious than people with (ex)partners. Further, women (*M*=7.07, *SD*=1.87) report to be more anxious than men (*M*=6.65, *SD*=2.02), *F* (2, 2028) = 11.97, *p*<.0001, η_p_^2^=.012. People who are (self) employed (M=6.93, SD=1.96), report similar levels of anxiety as people who are unemployed (*M*=6.77, *SD*=2.01), or students (M=6.59, SD=2.00), but more anxiety than retired people (M=6.31, SD=2.09) or *F* (3, 2027) = 3.69, *p*=.011, η_p_^2^=.005). Finally, people whose friends have become infected (either confirmed (*M*=7.37, *SD*= 1.76) or not confirmed (*M*=7.15, *SD*=1.76) were more anxious than people whose friends were not infected (not confirmed, *M*=6.82, *SD*=1.98, or confirmed, *M*=6.67, *SD*=2.01), *F* (3, 2027) = 14.57, *p*<.0001, η_p_^2^=.021).

**Appendix D**

Cross-country differences for the main dependent variables.

*Means (M) and Standard Deviations (SD), F-test (F) and Effect Size (η_p_^2^) of Scales per Country*

| **Scales** | **Germany** | | | **Spain** | | | **Netherlands** | | | | **UK** | | | ***F* (3, 2027)** | |  |  |
| --- | --- | --- | --- | --- | --- | --- | --- | --- | --- | --- | --- | --- | --- | --- | --- | --- | --- |
|  | | ***M***  ***(SD)*** |  | | ***M***  ***(SD)*** |  | | ***M***  ***(SD)*** |  | ***M***  ***(SD)*** | |  | ***F*** | | *η_p_^2^* | | |
| **Anxiety about Coronavirus** | | 6.26^c^  (2.16) |  | | 7.69^a^  (1.53) |  | | 6.39^c^  (1.81) |  | 7.12^b^  (1.92) | |  | 64.14*** | | .087 | |  |
| **Populist Attitudes** | | 4.72^a^  (.91) |  | | 4.88^b^  (.78) |  | | 4.40^c^  (.82) |  | 4.81^a^  (.81) | |  | 32.79*** | | .046 | |  |
| **Conspiracy Mentality** | | 4.13^a^  (1.20) |  | | 4.76^b^  (.93) |  | | 4.11^a^  (1.07) |  | 4.39^c^  (1.07) | |  | 39.77*** | | .056 | |  |
| **Anger at Government** | | 3.85^a^  (1.34) |  | | 4.62^b^  (1.45) |  | | 3.81^a^  (1.39) |  | 4.37^c^  (1.38) | |  | 41.71*** | | .058 | |  |
| **Approval of Hygiene Measures** | | 5.66^b^  (1.09) |  | | 6.17^a^  (.91) |  | | 5.49^b^  (.95) |  | 5.84^b^  (.98) | |  | 42.61*** | | .059 | |  |
| **Compliance with Hygiene Measures** | | 4.42^b^  (.87) |  | | 6.19^a^  (.86) |  | | 4.06^c^  (.72) |  | 4.04^c^  (.84) | |  | 771.82*** | | .533 | |  |
| **Anger at Transgressors** | | 4.11^a^  (1.06) |  | | 4.35^b^  (1.02) |  | | 4.12^a^  (.89) |  | 4.44^b^  (1.05) | |  | 13.292*** | | .019 | |  |

****p*<.001; different superscripts refer to significant differences at *p*<.01
